# Anti-Allergic Activity of Monoacylated Ascorbic Acid 2-Glucosides

**DOI:** 10.3390/molecules22122202

**Published:** 2017-12-12

**Authors:** Kaori Miura, Yuta Morishita, Hiroaki Matsuno, Yusuke Aota, Hideyuki Ito, Akihiro Tai

**Affiliations:** 1 Faculty of Life and Environmental Sciences, Prefectural University of Hiroshima, 5562 Nanatsuka-cho, Shobara, Hiroshima 727-0023, Japan; q531005dd@ed.pu-hiroshima.ac.jp (K.M.); yuta_morishita@icloud.com (Y.M.); q623021jw@ed.pu-hiroshima.ac.jp (H.M.); q723001ja@ed.pu-hiroshima.ac.jp (Y.A.); 2 Faculty of Health and Welfare Science, Okayama Prefectural University, 111 Kuboki, Soja, Okayama 719-1197, Japan; hito@fhw.oka-pu.ac.jp

**Keywords:** ascorbic acid derivatives, anti-allergic activity, degranulation, hyaluronidase, passive cutaneous anaphylaxis

## Abstract

2-*O*-α-d-Glucopyranosyl-l-ascorbic acid (AA-2G) is one of the stable ascorbic acid (AA) derivatives known as provitamin C agents. We have previously synthesized two types of monoacylated derivatives of AA-2G, 6-*O*-acyl-2-*O*-α-d-glucopyranosyl-l-ascorbic acids having a straight-acyl chain of varying length from C_4_ to C_18_ (6-sAcyl-AA-2G) and a branched-acyl chain of varying length from C_6_ to C_16_ (6-bAcyl-AA-2G) in order to improve the bioavailability of AA-2G. In this study, 6-sAcyl-AA-2G and 6-bAcyl-AA-2G *per se* showed the inhibitory effects on hyaluronidase activity and degranulation. 6-sAcyl-AA-2G exhibited strong inhibitory effects on hyaluronidase activity and degranulation in a concentration-dependent manner, and the inhibitory effects tended to become stronger with increasing length of the acyl chain. 2-*O*-α-d-Glucopyranosyl-6-*O*-hexadecanoyl-l-ascorbic acid (6-sPalm-AA-2G), which has a straight C_16_ acyl chain, was the most potent effective for inhibition of hyaluronidase activity and for inhibition of degranulation among the 6-sAcyl-AA-2G derivatives and the two isomers of 6-sPalm-AA-2G. Furthermore, percutaneous administration of 6-sPalm-AA-2G significantly inhibited IgE-mediated passive cutaneous anaphylaxis reaction in mice. These findings suggest that 6-sPalm-AA-2G will be useful for treatment of allergies.

## 1. Introduction

**Figure 1:**
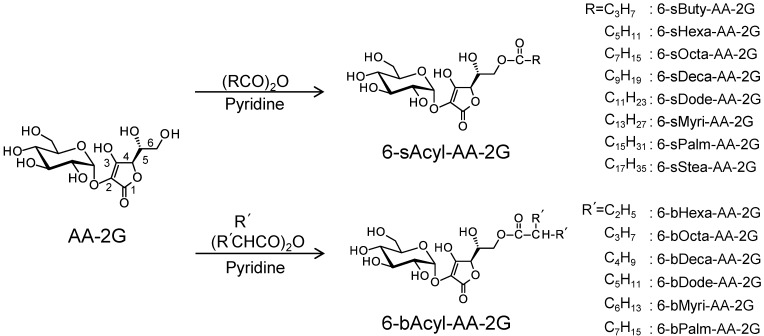
Structures of ascorbic acid (AA)-2G, 6-sAcyl-AA-2G and 6-bAcyl-AA-2G.

Ascorbic acid (AA), known as vitamin C, has various physiological and pharmacological activities such as antiscorbutic activity [1], anti-oxidation [2], enhancement of iron absorption [3] and drug metabolism [4]. However, AA is a very unstable molecule and is easily oxidized by exposure to heat, light, oxygen and moisture [5]. 2-*O*-α-d-Glucopyranosyl-l-ascorbic acid (AA-2G, Figure 1) is one of the stable ascorbate derivatives [6]. AA-2G shows vitamin C activity after enzymatic hydrolysis to AA by α-glucosidase, that is, AA-2G is a pro-vitamin C agent [7,8,9,10]. AA-2G has been permitted by the Japanese Government as a quasi-drug and food additive, and it is being used as a medical additive in the field of cosmetics. However, AA-2G has low skin permeability due to its high hydrophilicity [11]. In order to improve the bioavailability of AA-2G, we have synthesized two types of monoacylated derivatives of AA-2G: 6-*O*-acyl-2-*O*-α-d-glucopyranosyl-l-ascorbic acids having a straight-acyl chain of varying length from C_4_ to C_18_ (6-sAcyl-AA-2G, Figure 1) and having a 2-branched-acyl chain of varying length from C_6_ to C_16_ (6-bAcyl-AA-2G, Figure 1) [11,12]. 6-sAcyl-AA-2G and 6-bAcyl-AA-2G were shown to be stable in a neutral solution [11,12], and some of the monoacylated derivatives showed efficient physiological and pharmacological activities after enzymatic hydrolysis to AA by esterase and α-glucosidase [13,14,15]. Oral administration of 6-sAcyl-AA-2G and 6-bAcyl-AA-2G to scorbutic guinea pigs, as well as administration of AA-2G, ameliorated weight loss, and they also restored tissue AA levels more effectively than AA-2G [13,14]. 6-sAcyl-AA-2G and 6-bAcyl-AA-2G also induced greater enhancement of neurite outgrowth in PC12 cells than that induced by AA-2G at a low concentration [15]. Furthermore, several of the 6-sAcyl-AA-2G derivatives showed higher intestinal absorbability [13] and higher skin permeability [11,16] than those of AA-2G. These results indicated that 6-sAcyl-AA-2G and 6-bAcyl-AA-2G supplied AA efficiently and are superior to AA-2G in terms of bioavailability as provitamin C agents. 6-sAcyl-AA-2G and 6-bAcyl-AA-2G are the excellent pro-vitamin C agents that can be efficiently absorbed and metabolized. It was thought that AA-2G, 6-sAcyl-AA-2G and 6-bAcyl-AA-2G *per se* have no biological activity because they lose their activity by inhibiting the activity of α-glucosidase [15]. However, we found that AA-2G, 6-sAcyl-AA-2G and 6-bAcyl-AA-2G without being converted to AA exerted stronger anti-oxidant activity than that of AA stoichiometrically [17,18]. These results suggested that the derivatives *per se* have biological activity even before being converted to AA. If some kind of biological activity is observed by 6-sAcyl-AA-2G and 6-bAcyl-AA-2G, a new application of them can be achieved. We are now searching for pharmacologic activities of 6-sAcyl-AA-2G and 6-bAcyl-AA-2G.

In this study, to evaluate the anti-allergic activity of 6-sAcyl-AA-2G and 6-bAcyl-AA-2G, their hyaluronidase inhibitory activity and degranulation inhibitory activity were investigated. 2-*O*-α-d-Glucopyranosyl-6-*O*-hexadecanoyl-l-ascorbic acid (6-sPalm-AA-2G, Figure 1) showed the highest levels of both activities. We found the necessary structural moiety for inhibitory effects on hyaluronidase activity and degranulation by examining the activities of 6-sPalm-AA-2G and its isomers. The site of action in the degranulation inhibitory activity of 6-sPalm-AA-2G was also investigated. We report that 6-sPalm-AA-2G, which can be most expected as an anti-allergic drug in 6-sAcyl-AA-2G and 6-bAcyl-AA-2G, inhibited passive cutaneous anaphylaxis in the mouse ear.

## 2. Results and Discussion

Hyaluronidase is an enzyme decomposing hyaluronic acid and is found in the liver and testis of higher organisms. It is known to be related to an inflammatory response and degranulation [19,20]. Degranulation is a process that releases chemical mediators, such as histamine and leukotriene from mast cells, and is the cause of type I allergy [21,22]. Type I allergy, which is represented by pollen, asthma, etc., is caused by IgE-mediated release of chemical mediators from mast cells and basophils. It has been reported that a substance for inhibiting hyaluronidase activity has a degranulation inhibitory effect [19,23,24,25]. Thus, we evaluated the inhibitory effects of 6-sAcyl-AA-2G and 6-bAcyl-AA-2G on hyaluronidase activity and degranulation.

**Figure 2:**
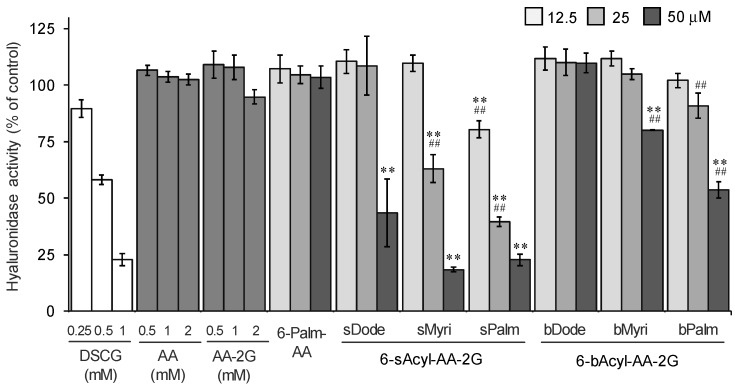
Inhibitory effects of 6-sAcyl-AA-2G and 6-bAcyl-AA-2G on hyaluronidase activity. All data represent means ± SD of three independent experiments. ** *p* < 0.01, vs. 6-sPalm-AA; ^##^ *p* < 0.01, 6-sDode-AA-2G vs. 6-sMyri-AA-2G and 6-sPalm-AA-2G, 6-bDode-AA-2G vs. 6-bMyri-AA-2G and 6-bPalm-AA-2G (Dunnett’s test).

First, the hyaluronidase inhibitory activities of 6-sAcyl-AA-2G and 6-bAcyl-AA-2G derivatives that have relatively long acyl-chains (C_12_ to C_16_), 6-sDode-, 6-sMyri-, 6-sPalm-, 6-bDode-, 6-bMyri- and 6-bPalm-AA-2G (Figure 1), were evaluated. Cromolyn sodium salt (DSCG), which is used clinically as an anti-allergy drug, was used as a positive control. As shown in Figure 2, 6-sAcyl-AA-2G and 6-bAcyl-AA-2G inhibited hyaluronidase activity in a concentration-dependent manner. 6-sMyri-AA-2G and 6-sPalm-AA-2G significantly inhibited the activity more than 6-sDode-AA-2G, and 6-bMyri-AA-2G and 6-bPalm-AA-2G significantly inhibited the activity more than 6-bDode-AA-2G, suggesting that hyaluronidase inhibitory activities increase with increasing length of their acyl group. Hyaluronidase inhibitory activities of 6-sDode-AA-2G at 50 μM, 6-sMyri-AA-2G at 25 and 50 μM and 6-sPalm-AA-2G at 12.5, 25 and 50 μM were significantly stronger than those of 6-bAcyl-AA-2G corresponding to the length of their acyl group (*t*-test, *p* < 0.01), suggesting that the activity is dependent on length of the main chain but not on the number of total carbon atoms. Of the 6-sAcyl-AA-2G derivatives, 6-sPalm-AA-2G, having a C_16_ straight-acyl chain, showed the highest inhibitory activity at a low concentration. 6-sPalm-AA-2G can be hydrolyzed to AA-2G, 6-*O*-palmitoyl-l-AA (6-Palm-AA) and AA with α-glucosidase and/or lipase. Interestingly, AA, AA-2G and 6-Palm-AA showed only slight activity as can be seen in Figure 2. AA and AA-2G did not show significant activity when their concentrations were increased to 10 mM (data not shown). It has been reported that 6-Palm-AA inhibits the activity of bovine testicular hyaluronidase [26]. In the present study, 6-Palm-AA tended to inhibit the activity of hyaluronidase when its concentration was higher than 50 μM (IC_50_ = 194 μM, data not shown). The 50% inhibitory concentration of 6-sPalm-AA-2G was 22 μM. Thus, 6-sPalm-AA-2G, which has a glucose moiety, exhibited a much stronger inhibitory effect than that of 6-Palm-AA, which does not have a glucose moiety. These results indicated that 6-sPalm-AA-2G *per se* showed hyaluronidase inhibitory activity and that the presence of glucose was important for exhibiting hyaluronidase inhibitory activity.

**Figure 3:**
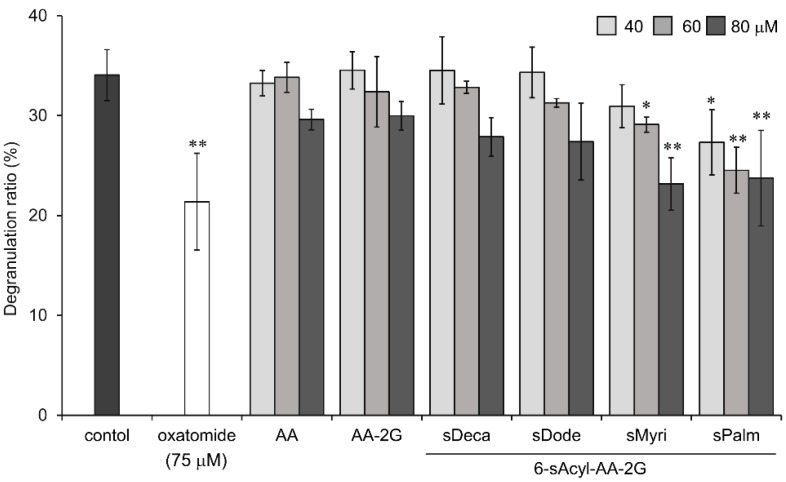
Inhibitory effects of 6-sAcyl-AA-2G on antigen-induced degranulation in RBL-2H3 cells. Anti-dinitrophenyl (DNP)-immunoglobulin E (IgE)-sensitized RBL-2H3 cells were incubated with the indicated samples and stimulated with DNP-human serum albumin (HSA). All data represent means ± SD of three independent cultures. * *p* < 0.05, ** *p* < 0.01 (Dunnett’s test) as compared with the control.

Next, we investigated the degranulation inhibitory activity of 6-sAcyl-AA-2G derivatives that have relatively long straight acyl-chains (C_10_ to C_16_) on degranulation in rat basophilic leukemia (RBL-2H3) cells. Oxatomide, which is used clinically as a chemical mediator release inhibitor, was used as a positive control. As shown in Figure 3, 6-sMyri- and 6-sPalm-AA-2G significantly inhibited degranulation in a concentration-dependent manner. AA showed very little activity. The activity of AA-2G was slightly dependent on the concentration, but no significant activity was observed even when the concentration of AA-2G was increased (data not shown). As with the hyaluronidase inhibitory activity, degranulation inhibitory activities of 6-sAcyl-AA-2G samples tended to increase with increasing length of their acyl group. 6-sPalm-AA-2G suppressed the degranulation most strongly among the derivatives, and the activity of 6-sPalm-AA-2G was comparable to that of oxatomide. These results suggest that 6-sPalm-AA-2G is the most effective for inhibition of degranulation activity as well as inhibition of hyaluronidase activity.

**Figure 4:**
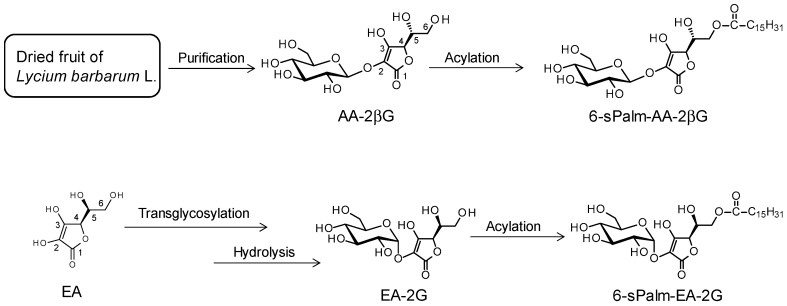
Structures and scheme for synthesis of 6-sPalm-AA-2βG and 6-sPalm-EA-2G.

We then synthesized two isomers of 6-sPalm-AA-2G for studies of structure-activity relationships in hyaluronidase inhibitory activity and degranulation inhibitory activity. To evaluate the influence of the steric configuration of the glycosidic bond, one of the isomers of 6-sPalm-AA-2G, 2-*O*-β-d-glucopyranosyl-6-*O*-hexadecanoyl-l-ascorbic acid (6-sPalm-AA-2βG, Figure 4), which has a β-glycosidic bond, was synthesized. 6-sPalm-AA-2βG was synthesized by the following steps: extraction and purification of AA-2βG from dried fruit of *Lycium barbarum* [27] and acylation of AA-2βG. The other isomer is 2-*O*-α-d-glucopyranosyl-6-*O*-hexadecanoyl-d-erythorbic acid (6-sPalm-EA-2G, Figure 4), which differs in the configuration at the C-5 position of the AA moiety. 6-sPalm-EA-2G was synthesized by the following steps: glycosylation of erythorbic acid and acylation of erythorbic acid 2-glucoside.

**Figure 5:**
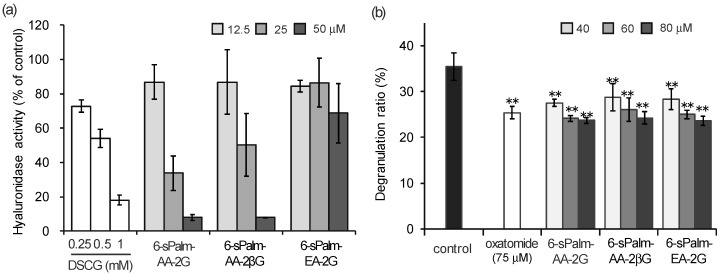
Inhibitory effects of 6-sPalm-AA-2G and its isomers on hyaluronidase activity (**a**) and antigen-induced degranulation (**b**). All data represent means ± SD of three independent experiments. ** *p* < 0.01 (Dunnett’s test) as compared with the control.

In order to perform studies of structure–activity relationships in the hyaluronidase inhibition activity and degranulation inhibitory activity, the activities of 6-sPalm-AA-2G and its isomers were evaluated. First, the hyaluronidase inhibitory activities of the isomers were evaluated. 6-sPalm-AA-2βG significantly inhibited the hyaluronidase activity (Figure 5a). The inhibitory concentrations at 50% of 6-sPalm-AA-2G and 6-sPalm-AA-2βG were 21 μM and 25 μM, respectively, and there was no significant difference. As shown in Figure 2, it is important that glucose is bonded to 6-sPalm-AA to exhibit hyaluronidase inhibitory activity. However, there was no difference in hyaluronidase inhibitory activity between the two types of glycosidic bonds. Interestingly, 6-sPalm-EA-2G showed only slight activity. These results indicated that the stereostructure of the hydroxyl group at the C-5 position of the AA moiety was more important than that of the glucosyl group at the C-2 position for exhibiting hyaluronidase inhibitory activity. Next, the degranulation inhibitory effects of 6-sPalm-AA-2G and the two isomers were investigated. The two isomers showed the same level anti-degranulation activity as that of 6-sPalm-AA-2G, suggesting that the activity was not influenced by the stereostructures of the glucosyl group at the C-2 position and the hydroxyl group at the C-5 position of the AA moiety (Figure 5b). These results showed that 6-sPalm-AA-2G was the most effective for inhibition of hyaluronidase activity and for inhibition of degranulation among the 6-sAcyl-AA-2G derivatives and the two isomers of 6-sPalm-AA-2G.

**Figure 6:**
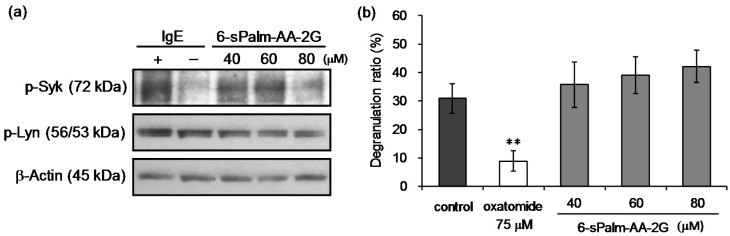
Effects of 6-sPalm-AA-2G on the signaling pathway leading to degranulation in RBL-2H3 cells. (**a**) Antigen-induced phosphorylation of Lyn and Syk kinases in RBL-2H3 cells; (**b**) Calcium ionophore A23187-induced degranulation. All data represent means ± SD of three independent cultures. ** *p* < 0.01 (Dunnett’s test) as compared with the control.

It is important for type I allergy therapy to suppress degranulation reaction. The antigen-induced degranulation in RBL-2H3 cells is caused by aggregation of FcεRI on the surface of cells, phosphorylation of tyrosine, activation of phosphoinositide 3-kinase and elevation of intracellular Ca^2+^ level. Calcium ionophore stimulation, which increases intracellular Ca^2+^ levels, also causes degranulation [28,29]. To reveal the mechanism for the inhibitory effect of 6-sPalm-AA-2G on degranulation in RBL-2H3 cells, we examined the inhibitory effects of 6-sPalm-AA-2G on phosphorylation of signaling tyrosine kinases (Lyn and Syk) induced by antigen stimulation and on degranulation by calcium ionophore A23187 stimulation. First, we examined the effect of 6-sPalm-AA-2G on phosphorylation of Lyn and Syk, which play important roles in early signaling events in antigen-induced degranulation. 6-sPalm-AA-2G inhibited the expression of phosphorylated Syk in a concentration-dependent manner (Figure 6a). Phosphorylated Lyn expression was not inhibited. Next, the inhibitory effect of 6-sPalm-AA-2G on calcium ionophore A23187-induced degranulation was evaluated. As shown in Figure 6b, 6-sPalm-AA-2G slightly increased calcium ionophore A23187-stimulated degranulation but there was no statistically significant difference between control and treatment groups. These results suggested that 6-sPalm-AA-2G suppressed the degranulation by inhibiting the upstream region of a signal transmission pathway of antigen-induced degranulation, while it had no effect on calcium channels expressed on the cell membranes of RBL-2H3 cells and on the increase of intracellular calcium.

**Figure 7:**
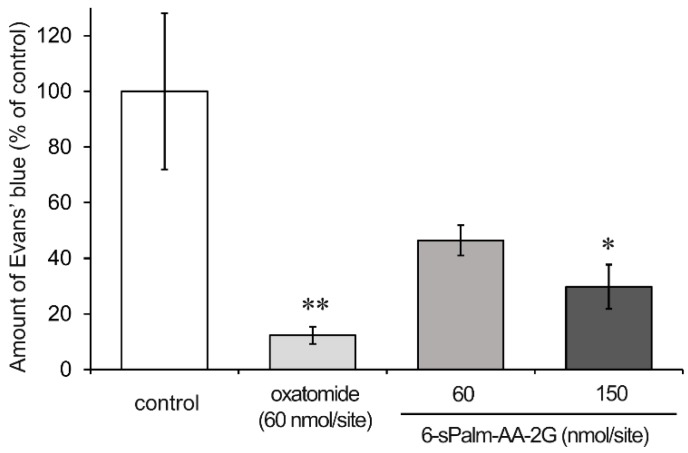
Inhibitory effect of 6-sPalm-AA-2G on PCA reaction in mice. Mice were percutaneously administered the indicated samples: control (*n* = 7), oxatomide (*n* = 5), 6-sPalm-AA-2G 60 nmol/site (*n* = 4), 150 nmol/site (*n* = 5). All data represent means ± SE. * *p* < 0.05, ** *p* < 0.01 (Dunnett’s test) as compared with the control.

Finally, we investigated the effects of 6-sPalm-AA-2G on IgE-mediated passive cutaneous anaphylaxis (PCA) reaction using ICR mice. In the case of oral administration, 6-sAcyl-AA-2G was rapidly hydrolyzed to AA in the intestine without being absorbed as the intact form [11,16]. Thus, only AA concentration in the plasma was increased after oral administration of 6-sAcyl-AA-2G. In experiment using a skin model, some of 6-sAcyl-AA-2G permeated the skin as the intact form, while some was hydrolyzed [11,16]. Thus, we decided to use percutaneous administration of 6-sPalm-AA-2G to evaluate its inhibitory activity on PCA reaction. Ears of mice were sensitized with anti-dinitrophenyl (DNP)-immunoglobulin E (IgE) for 24 h, and then test samples were applied to the ears. The mice were then challenged with DNP-human serum albumin (HSA) including 1% Evans’ blue. Evans’ blue dye extravasation (% of control) of oxatomide at 60 nmol/site in the ears was 12.3%, and the percentages for 6-sPalm-AA-2G at 60 and 150 nmol/site were 46.4% and 29.7%, respectively (Figure 7). 6-sPalm-AA-2G significantly inhibited the PCA reaction of the mouse ear in a concentration-dependent manner. These results suggested that 6-sPalm-AA-2G penetrated through the skin and showed inhibitory activity against PCA reaction.

We found in the present study that intact 6-sPalm-AA-2G had inhibitory effects on hyaluronidase activity, degranulation inhibitory activity and inhibitory activity on PCA reaction, suggesting that 6-sPalm-AA-2G would be useful for treatment of allergies. To our knowledge, there is no report on the anti-allergic activity of AA derivatives. It has been reported that AA decomposed histamine in the presence of Cu^2+^ in vitro [30] and that intravenous infusion of AA decreased serum histamine concentrations in patients with allergic diseases [31]. Thus, 6-sPalm-AA-2G is expected to exert further anti-allergic activity as AA after being hydrolyzed to AA. It can be expected that 6-sPalm-AA-2G *per se* will show anti-allergic activity via hyaluronidase inhibitory activity and degranulation inhibitory activity by using it in the form of, e.g., ointment, liniment, inhalant, collunarium, or eye drops and that after being hydrolyzed to AA, it will exhibit further anti-allergic activity and vitamin C activity as AA.

## 3. Materials and Methods

### 3.1. General Methods

AA-2G was provided by Hayashibara Biochemical Laboratories (Okayama, Japan). A series of stable lipophilic vitamin C derivatives, 6-sAcyl-AA-2G and 6-bAcyl-AA-2G, were synthesized in our laboratory as described previously [11,12]. Sodium ascorbate, oxatomide, hyaluronic acid sodium salt from a rooster comb and *p*-dimethylaminobenzaldehyde were acquired from Wako Pure Chemical Industries, Osaka, Japan. Dulbecco’s modified Eagle’s medium, monoclonal anti-dinitrophenyl antibody (DNP-IgE), dinitrophenyl-labeled human serum albumin (DNP-HSA), cromolyn sodium salt (DSCG), Triton X-100, hyaluronidase type IV-S from a bovine testis and compound 48/80 were purchased from Sigma-Aldrich Co., St. Louis, MO, USA. Fetal bovine serum was acquired from HyClone, Logan, UT, USA. Penicillin–streptomycin mixed solution and *p*-nitrophenyl-2-acetamido-2-deoxy-β-d-glucopyranoside were obtained from Nacalai Tesque, Kyoto, Japan. Anti-phospho-Syk (Tyr525/526) rabbit monoclonal antibody, anti-phospho-Lyn (Tyr507) rabbit polyclonal antibody and horseradish peroxidase-conjugated anti-rabbit IgG (Goat) were purchased from Cell Signaling Technology, Danvers, MA, USA. All of the chemicals used were of the highest grade commercially available. TOYOPEARL HW-40C (Tosoh Corporation, Tokyo, Japan), Chromatorex ODS (Fuji Silysia Chemical, Aichi, Japan), Wakogel C-200 (Wako Pure Chemical Industries) and activated charcoal (Wako Pure Chemical Industries) were used for column chromatography. NMR spectra were obtained on a Varian NMR System 600 MHz instrument. The values of chemical shifts are expressed in ppm, and each coupling constant (J) is expressed in Hz. Electron spray ionization (ESI) high-resolution mass sptectra were recorded on a Bruker Daltonics MicrOTOF II instrument using direct sample injection. The purity of 6-sPalm-AA-2βG and that of 6-sPalm-EA-2G were assessed by HPLC and were found to be higher than 95%. HPLC analysis was performed on an Inertsil Ph-3 column (3 μm, φ 4.6 mm × 100 mm, GL Sciences Inc., Tokyo, Japan) with methanol/water/formic acid at 65:34.9:0.1 (*v*/*v*/*v*) at a flow rate of 0.7 mL/min. UV detection was performed at 254 nm.

### 3.2. Synthesis of 2-O-β-d-Glucopyranosyl-6-O-hexadecyl-l-ascorbic Acid 

2-*O*-β-d-Glucopyranosyl-6-*O*-hexadecyl-l-ascorbic acid (AA-2βG) was obtained from dried fruit of *Lycium barbarum* L. [27] Finely chopped dried fruit of *Lycium barbarum* L. (504.75 g, Nihon Health Corporation, Yokohama, Japan) was extracted with 4 L of 60% MeOH at room temperature for 5 days. The extract was filtered and evaporated to dryness, and the residue (231.16 g) was chromatographed on an activated charcoal column eluted with a stepwise gradient of MeOH–H_2_O containing a 0.5% formic acid solvent system (0%, 10%, 20%, 30%, 50% and 70%, *v*/*v*). Fractions eluted by 50% and 70% MeOH (9.70 g) were again chromatographed on an activated charcoal column eluted with a stepwise gradient of 20%, 30%, 40%, 50%, 60% and 70%, *v*/*v*. Fractions eluted by 50%, 60% and 70% MeOH (4.98 g) were further chromatographed on a TOYOPEARL HW-40C column (φ 4.0 × 39.0 cm) eluted with 480 mL of a 0.5% formic acid aqueous solution to give 80 fractions. Fractions 56–69 (3.20 g) were again chromatographed on a TOYOPEARL HW-40C column (φ 4.0 × 39.0 cm) with the same conditions to give 80 fractions. Fractions 56–69 (3.00 g) were then chromatographed on a Chromatorex ODS column (φ 3.0 × 49.0 cm) eluted with 350 mL of a 0.5% formic acid aqueous solution to give 75 fractions. Fractions 31–75 were mixed and concentrated to give AA-2βG (2.31 g). AA-2βG was acylated according to our previous study [11]. AA-2βG (0.6 g, 1.77 mmol) and palmitic anhydride (1.05 g, 2.12 mmol) were dissolved in pyridine (6.0 mL), and the mixture was stirred at 60 °C for 30 min. The reaction mixture was then evaporated to dryness. The resulting residue was chromatographed on a Wakogel C-200 column eluted with a stepwise gradient of MeOH–EtOAc containing a 0.1% formic acid solvent system (0%, 10%, 20% and 30%, *v*/*v*). Fractions eluted by 20% and 30% MeOH (0.44 g) were further chromatographed on a TOYOPEARL HW-40C eluted with 450 mL of 70% MeOH–H_2_O containing a 0.1% formic acid to give 75 fractions. Fractions 57–72 (0.41 g) were then chromatographed on a Chromatorex ODS column eluted with a stepwise gradient of MeOH–H_2_O containing a 0.1% formic acid solvent system (75%, 77.5%, 80%, 82.5%, 85% and 87.5%, *v*/*v*). Fractions eluted by 85% and 87.5% MeOH were mixed and concentrated to give 6-sPalm-AA-2βG (320.1 mg, yield 32.3%). ^1^H NMR (CD_3_OD, 600 MHz) δ_H_: 0.94 (3H, t, *J* = 7.2 Hz), 1.33 (24H, m), 1.67 (2H, quint, *J* = 7.2 Hz), 2.42 (2H, t, *J* = 7.5 Hz), 3.30 (4H, m, 2′,3′,6′-H), 3.73 (1H, dd, *J* = 5.4, 12.0 Hz, 5′-H), 3.89 (1H, m, 4′-H), 4.17 (1H, m, 5-H), 4.22 (1H, m, 6-Ha), 4.31 (1H, dd, 6.6, 10.8 Hz, 6-Hb), 4.85 (1H, d, *J* = 7.2 Hz, 1′-H), 4.88 (1H, d, *J* = 1.8 Hz, 4-H). ^13^C NMR (CD_3_OD, 150 MHz): δ_C_ 13.01, 22.31, 24.64, 28.76, 29.04, 29.16, 29.28, 29.33 (×4), 29.34 (×2), 31.64, 33.43, 60.77, 64.13, 66.46, 69.59, 73.16, 75.99, 76.05, 76.88, 102.78, 118.29, 161.304, 171.02, 173.66. ^1^H-^1^H COSY and HMBC spectrum data are shown in Supporting information (Figures S1, S2, S5 and S7). ESI-HRMS [M − H]^−^: calcd. for C_28_H_47_O_12_: 575.3073, found 575.3084. HPLC: r.t. 12.57 min, 98.6% purity.

### 3.3. Synthesis of 2-O-α-d-Glucopyranosyl-6-O-hexadecyl-d-erythorbic Acid 

Erythorbic acid 2-glucoside (EA-2G) was prepared by the modified method described in our previous report [32]. Briefly, the process for synthesis of EA-2G consisted of two steps: transglycosylation by cyclodextrin glucanotransferase from *Thermoanaerobacter* sp. with starch and hydrolysis by amyloglucosidase from *Aspergillus niger*. EA-2G was acylated according to our previous study [11]. EA-2G (1.0 g, 2.96 mmol) and palmitic anhydride (1.76 g, 3.55 mmol) were dissolved in pyridine (10 mL), and the mixture was stirred at 60 °C for 30 min. The reaction mixture was then evaporated to dryness. The resulting residue was chromatographed on a TOYOPEARL HW-40C column with a stepwise gradient of MeOH–H_2_O containing a 0.1% formic acid solvent system (80% and 90%, *v*/*v*). Fractions mainly containing acylated EA-2G (1.15 g) were concentrated and recrystallized from ethanol to give 6-sPalm-EA-2G (544.3 mg, yield 31.8%). ^1^H NMR (CD_3_OD, 600 MHz) δ_H_: 0.94 (3H, t, *J* = 7.2 Hz), 1.33 (24H, m), 1.66 (2H, quint, *J* = 7.2 Hz), 2.39 (2H, t, *J* = 7.5 Hz), 3.57 (1H, t, *J* = 9.6 Hz, 4′-H), 3.79 (4H, m, 2′, 3′, 6′-H), 4.07 (1H, ddd, *J* = 2.4, 4.8, 9.6 Hz, 5′-H), 4.22 (1H, m, 5-H), 4.25 (2H, m, 6-H), 4.91 (1H, overlapped with solvent, 4-H), 5.41 (1H, d, *J* = 3.6 Hz, 1′-H). ^13^C NMR (CD_3_OD, 150 MHz): δ_C_ 13.01, 22.31, 24.51, 28.77, 28.99, 29.04, 29.18, 29.30, 29.36 (×5), 31.64, 33.43, 60.65, 63.32, 68.47, 69.59, 71.94, 72.93, 73.46, 76.68, 100.42, 118.95, 161.11, 170.49, 173.77. ^1^H-^1^H COSY and HMBC spectrum data are shown in Supporting information (Figures S3, S4, S6 and S8). ESI-HRMS [M − H]^−^: calcd. for C_28_H_47_O_12_: 575.3073, found 575.3092. HPLC: r.t. 11.65 min, 97.5% purity. 

### 3.4. Evaluation of Hyaluronidase Inhibitory Activity

The effects of samples on hyaluronidase activity were determined by a modification of the method of Morgan–Elson [33]. Samples (each 20 μL) dissolved in 0.1 M acetate buffer (pH 4.0) containing 30% DMSO and hyaluronidase dissolved in the buffer (1200 units/mL, 50 μL) were incubated at 37 °C for 20 min. Then, compound 48/80, CaCl_2_ and NaCl (10 μL each) were added and the mixture was incubated at 37 °C. After 20 min, hyaluronic acid (1.2 mg/mL, 50 μL) was added and the mixture was incubated at 37 °C for 40 min. Then the reaction was stopped by adding 0.4 N NaOH (40 μL) and cooling it on ice. After 10 min, 800 mM sodium borate buffer (pH 9.1, 30 μL) was added, and the mixture was incubated at 100 °C for 3 min and cooled on ice. Aliquots of 30 μL of the mixture were transferred to a 96-well microplate, and 150 μL of a coloring reagent (100 mg of *p*-dimethylbenzaldehyde dissolved in 10 mL of acetic acid solution containing 125 μL of concentrated HCl) was added. The mixture was then incubated at 37 °C for 20 min. The absorbance was measured at 585 nm, and hyaluronidase inhibitory activity (%) was quantified.

### 3.5. Antigen-Mediated Degranulation Assay

RBL-2H3 cells were purchased from the JCRB Cell Bank (Osaka, Japan). The cells were grown in Dulbecco’s modified Eagle’s medium containing 10% heat-inactivated fetal bovine serum, 100 U/mL of penicillin, and 100 μg/mL of streptomycin in a humidified atmosphere of 5% CO_2_ at 37 °C. The cells were cultured in a 96-well plate (5.0 × 10^4^ cells/200 μL/well) for 24 h at 37 °C and incubated in a growth medium containing 50 ng/mL of anti-DNP-IgE for 2 h. Then the cells were washed with modified Tyrode’s (MT) buffer, and 90 μL of each of the test samples or oxatomide (75 μM) dissolved in MT buffer containing 0.25% dimethyl sulfoxide was added. After 20 min of incubation, 10 μL of DNP-HSA (final concentration of 50 ng/mL) was added to the cells and the culture was incubated for 1 h. The supernatant was collected, and the cells were lysed with MT buffer containing 0.1% Triton X-100. The β-hexosaminidase activities of the supernatant and cell lysate were measured by the method of Demo et al. [34]. The supernatant or the cell lysate (20 μL) was mixed with 3.3 mM *p*-nitrophenyl-2-acetamide-2-deoxy-β-d-glucopyranoside (40 μL) in 100 mM citrate buffer (pH 4.5), and the mixture was incubated in a 96-well plate at 37 °C for 90 min. The reaction was terminated by adding 2 M glycine buffer (pH 10.4, 40 μL), and the absorbance at 405 nm was measured using a microplate reader (Varioskan FC from Thermo Fisher Scientific, Waltham, MA, USA). 

### 3.6. Calcium Ionophore-Mediated Degranulation Assay

RBL-2H3 cells were seeded into a 96-well microplate at a cell density of 5 × 10^4^ cells and incubated at 37 °C for 24 h. Then the cells were washed with MT buffer and incubated with 90 μL of each of the test samples dissolved in MT buffer containing 0.25% dimethyl sulfoxide. After 20 min, 10 μL of the calcium ionophore A23187 (100 μM) was added to each well and incubated at 37 °C for 1 h. The following experimental procedures were the same as those described above.

### 3.7. Immunoblotting Analysis

RBL-2H3 cells were seeded into a 60 mm culture dish at 1.0 × 10^6^ cells/5 mL/dish and treated with anti-DNP-IgE for sensitization, followed by treatment with samples and stimulation with DNP-HSA. Fifteen minutes after the stimulation, the cells were washed and lysed using lysis buffer. Cell lysates were subjected to sodium dodecyl sulfate-polyacrylamide gel electrophoresis (12.5%) and then transferred onto polyvinylidenefluoride membranes. After blocking for 1 h in blocking buffer (EzBlock Chemi, ATTO Corporation, Tokyo, Japan), the membranes were incubated with a primary antibody at 4 °C overnight, followed by incubation with a horseradish peroxidase-conjugated secondary antibody at room temperature for 1 h. The proteins were detected with an enhanced ECL kit (Thermo Fisher Scientific, Waltham, MA, USA) and were visualized by exposing the membrane to a medical X-ray film (Fujifilm Corporation, Tokyo, Japan) in a dark room. 

### 3.8. PCA Reaction in Mice

Five-week-old male ICR mice were obtained from CLEA Japan, Inc. (Tokyo, Japan) and maintained at a room temperature of 23 ± 5 °C. The experiments were approved by the Committee for Ethics in Animal Experiments of the Prefectural University of Hiroshima. An IgE-induced passive cutaneous anaphylaxis (PCA) reaction was carried out as follows. Each mouse (7 Ws) was intradermally injected with 20 μL of anti-DNP-IgE antibody (5 mg/mL) in the right-side ear and the same volume of saline was injected in the left-side ear. After 24 h, 30 μL of each solution of oxatomide at 2 mM (60 nmol/site) and 6-sPalm-AA-2G at 2 and 5 mM (60 and 150 nmol/site, respectively) was applied to the right and left ears. 6-sPalm-AA-2G and oxatomide were all dissolved in ethanol/glycerin/Tween-20/H_2_O = 20/5/0.5/74.5 (*v*/*v*/*v*/*v*). After 3.5 h, the mice received an intravenous injection of saline containing DNP-HSA (0.4 mg/mL, 250 μL) and 1% Evan’s blue. After 30 min, the mice were sacrificed by cervical dislocation and their ears were removed. Each ear was immersed in 500 μL of 1 N KOH solution and dissolved overnight at room temperature. The extravasated Evan’s blue dye was extracted with acetone-0.3 M phosphoric acid (13:5) solution and centrifuged for 20 min at 700× *g*. Absorbance was measured (620 nm), and the percentage of inhibitory effect on PCA reaction was calculated by subtracting the absorbance of right ear from that of left ear.

### 3.9. Data Analysis

In vitro results are expressed as means and SD and in vivo results are expressed as means and SE. Comparison of two means was performed by *t*-test. Multiple data comparisons were performed by analysis of variance followed by Dunnett’s test (* *p* < 0.05, ** *p* < 0.01).

## 4. Conclusions

Some of 6-sAcyl-AA-2G and 6-bAcyl-AA-2G show efficient vitamin C activity after enzymatic hydrolysis to AA by esterase and α-glucosidase; that is, they are excellent pro-vitamin C agents. In this study, we found that 6-sAcyl-AA-2G and 6-bAcyl-AA-2G showed strong inhibitory effects on hyaluronidase activity and degranulation by themselves. Study of the structure-activity correlation in the hyaluronidase inhibitory effect and anti-degranulation effect of 6-sAcyl-AA-2G, 6-bAcyl-AA-2G and isomers of 6-sPalm-AA-2G showed that 6-sPalm-AA-2G was a potent inhibitor of hyaluronidase activity and degranulation. Percutaneous administration of 6-sPalm-AA-2G significantly suppressed PCA reaction, which is a model of type I allergy. 6-sPalm-AA-2G is expected to exert anti-allergic activity not only as the intact form but also as AA, which is a hydrolysate of 6-sPalm-AA-2G. Our results provide evidence that 6-sPalm-AA-2G is a potent drug candidate for the therapy of type I allergies.

## Supplementary Materials

Supplementary materials are available online.
