# Supplementary material for: Anti-Allergic Activity of Monoacylated Ascorbic Acid 2-Glucosides
Source: Molecules. 2017 Dec 12;22(12):2202. doi: 10.3390/molecules22122202 (PMC6149712; doi:10.3390/molecules22122202)
Supplement: Supplementary file 1 [file molecules-22-02202-s001.pdf]

Supplementary Materials

Anti-allergic activity of monoacylated ascorbic acid 2-glucosides

Kaori Miura [1], Yuta Morishita [1], Hiroaki Matsuno [1], Yusuke Aota [1], Hideyuki Ito [2], Akihiro Tai [1,*]

[1]*Faculty of Life and Environmental Sciences, Prefectural University of Hiroshima, 5562 Nanatsuka-cho, Shobara, Hiroshima 727-0023, Japan*

[2]*Faculty of Health and Welfare Science, Okayama Prefectural University, 111 Kuboki, Soja, Okayama 719-1197, Japan*

Contents:

Figure S1. ^{1}H NMR spectrum of 6-sPalm-AA-2βG

Figure S2. ^{1}C NMR spectrum of 6-sPalm-AA-2βG

Figure S3. ^{1}H NMR spectrum of 6-sPalm-EA-2G

Figure S4. ^{1}H NMR spectrum of 6-sPalm-EA-2G

Figure S5. HMBC spectrum of 6-sPalm-AA-2βG

Figure S6. HMBC spectrum of 6-sPalm-EA-2G

Figure S7. ^{1}H-^{1}H COSY spectrum of 6-sPalm-AA-2βG

Figure S8. ^{1}H-^{1}H COSY spectrum of 6-sPalm-EA-2G

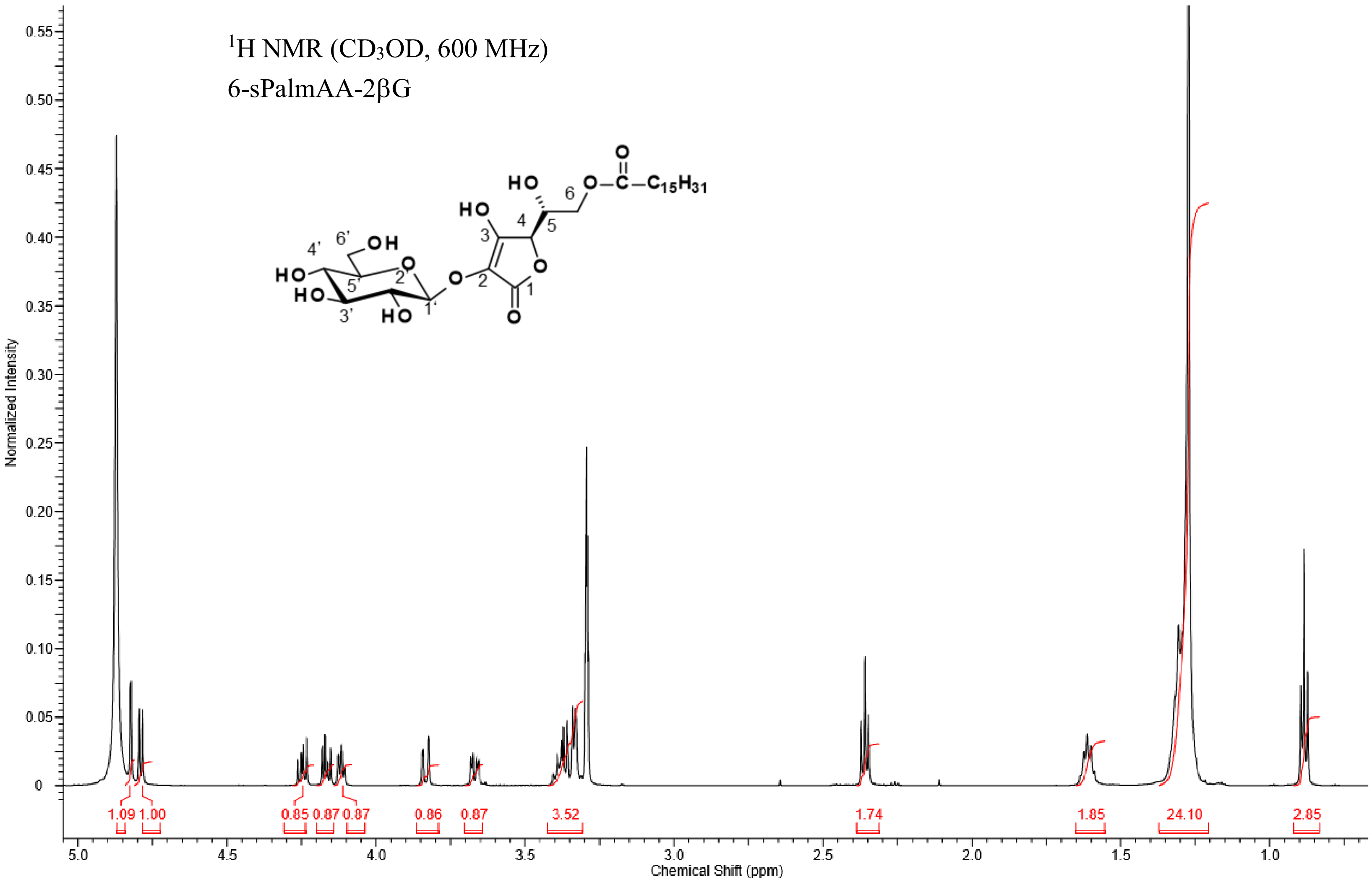

Figure S1. ^{1}H spectrum of 6-sPalm-AA-2βG

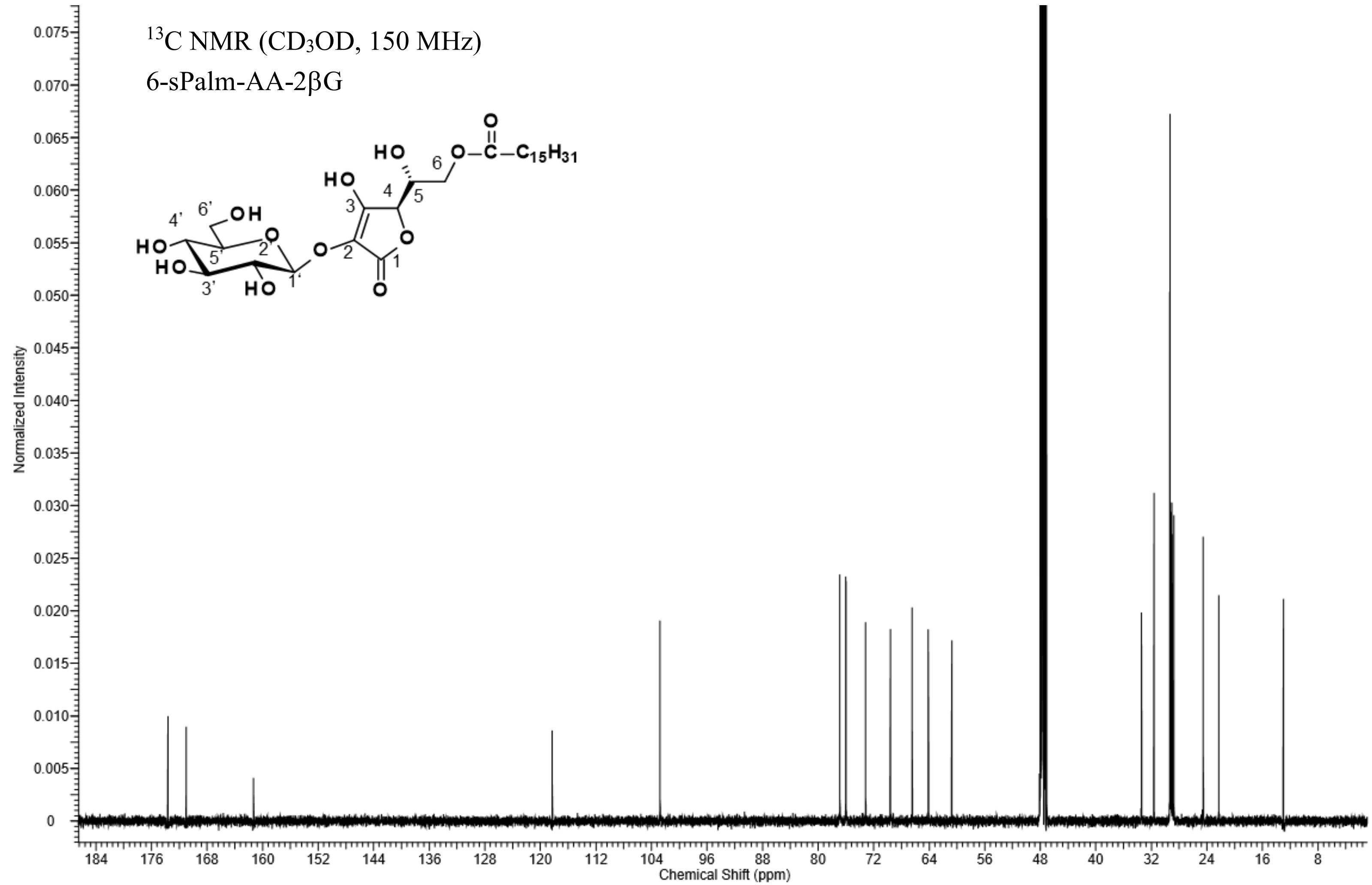

Figure S2. ^{13}C NMR spectrum of 6-sPalm-AA-2βG

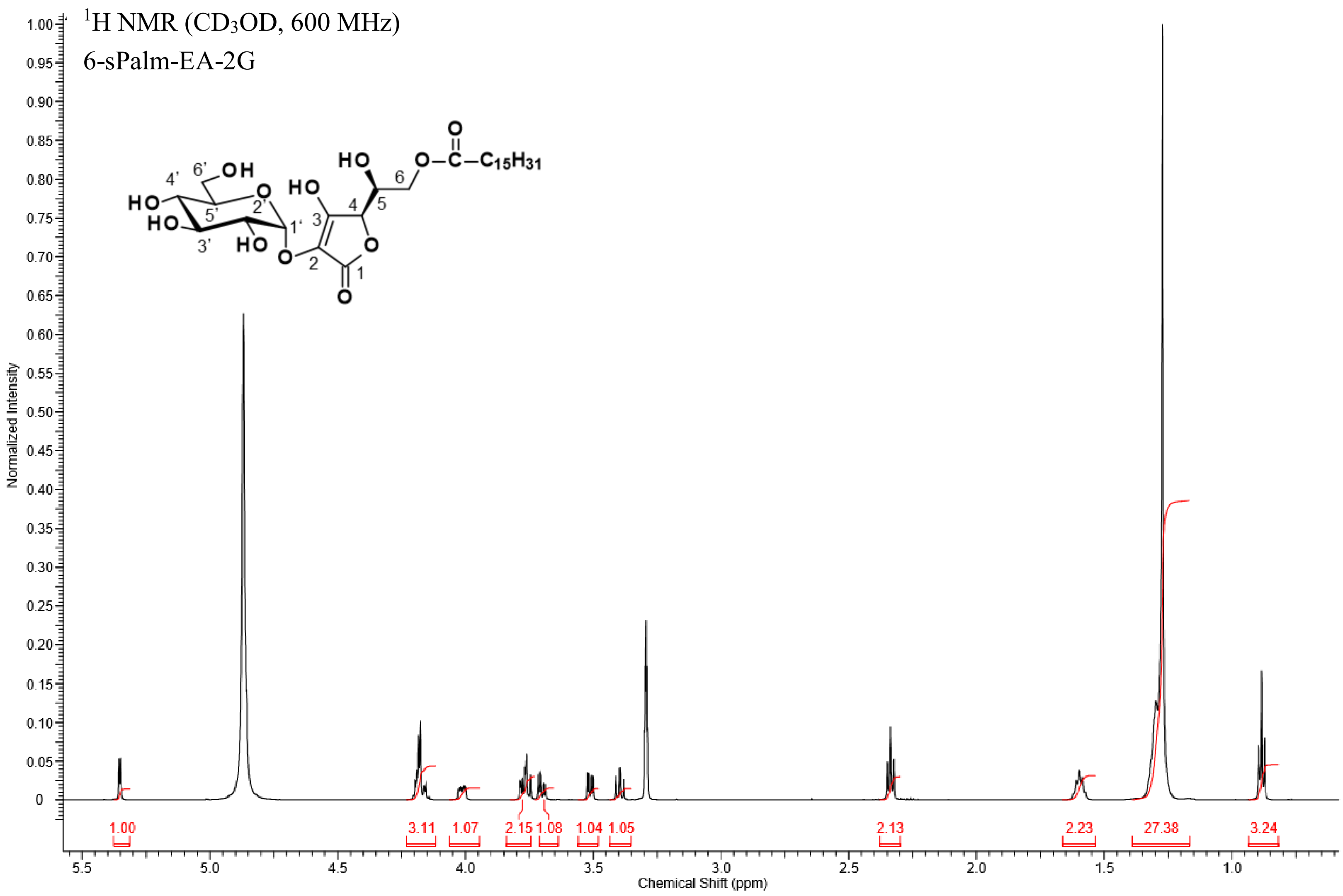

Figure S3. ^{1}H spectrum of 6-sPalm-EA-2G

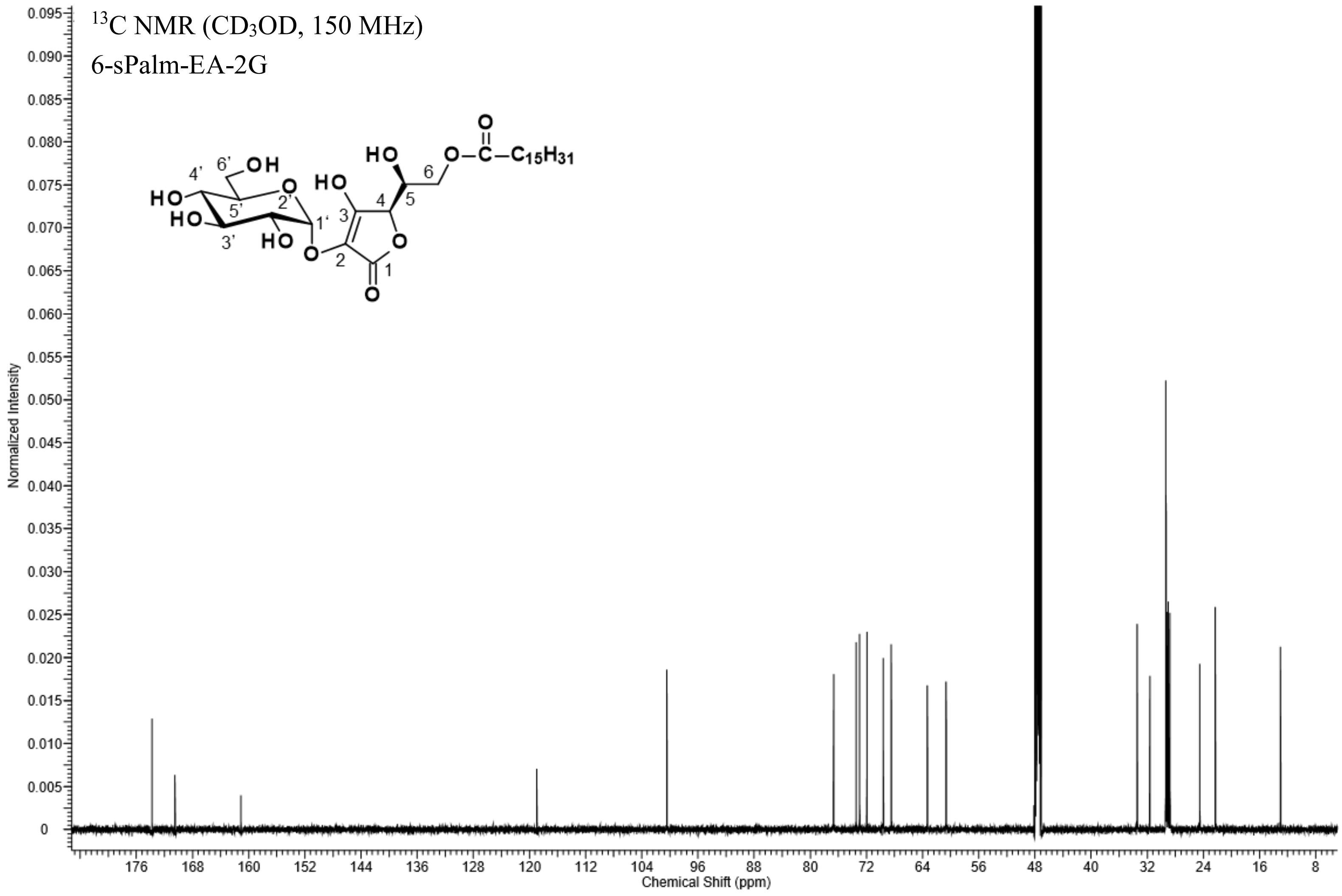

Figure S4. ^{13}C NMR spectrum of 6-sPalm-EA-2G

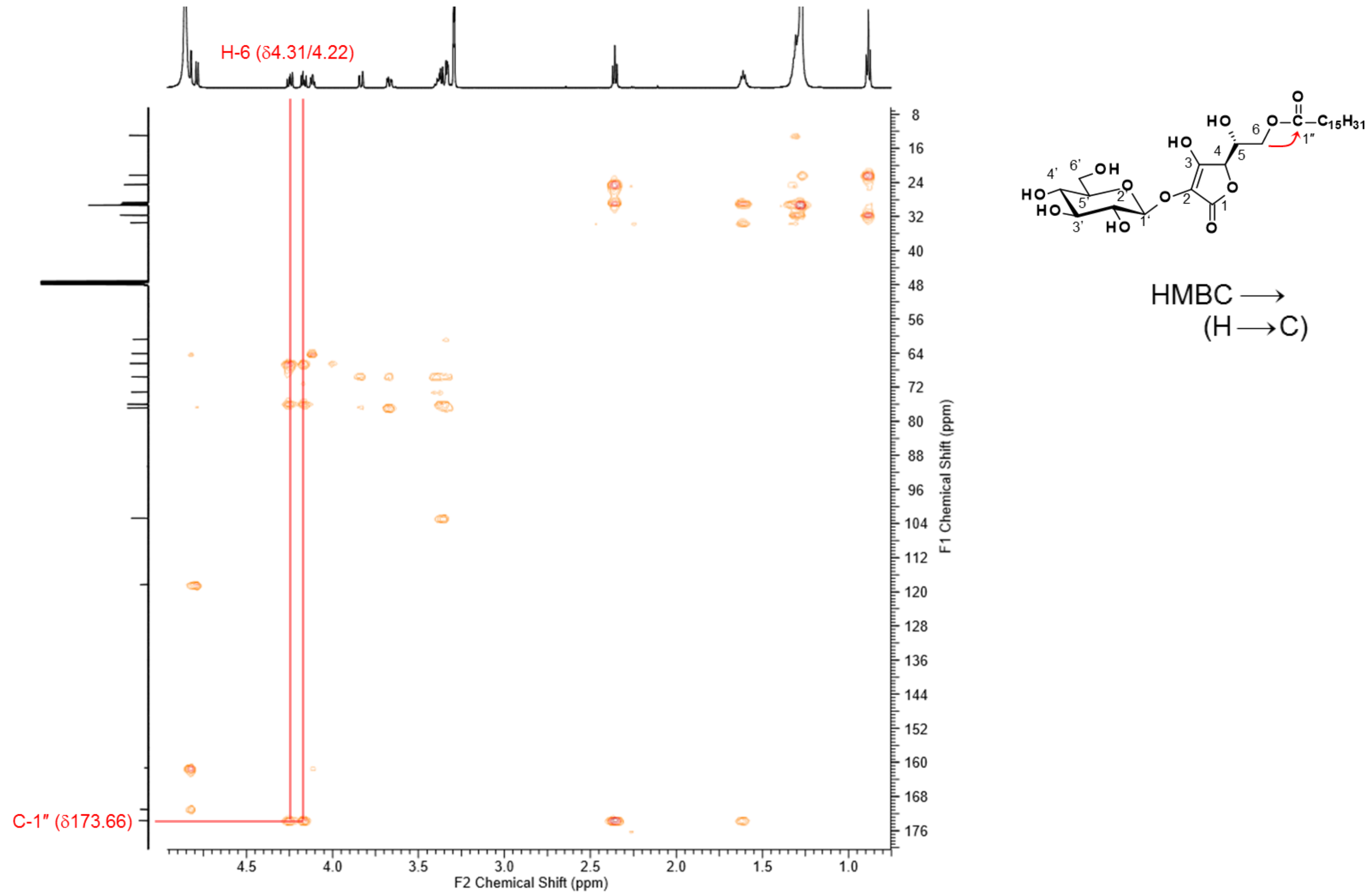

Figure S5. HMBC spectrum of 6-sPalm-AA-2βG

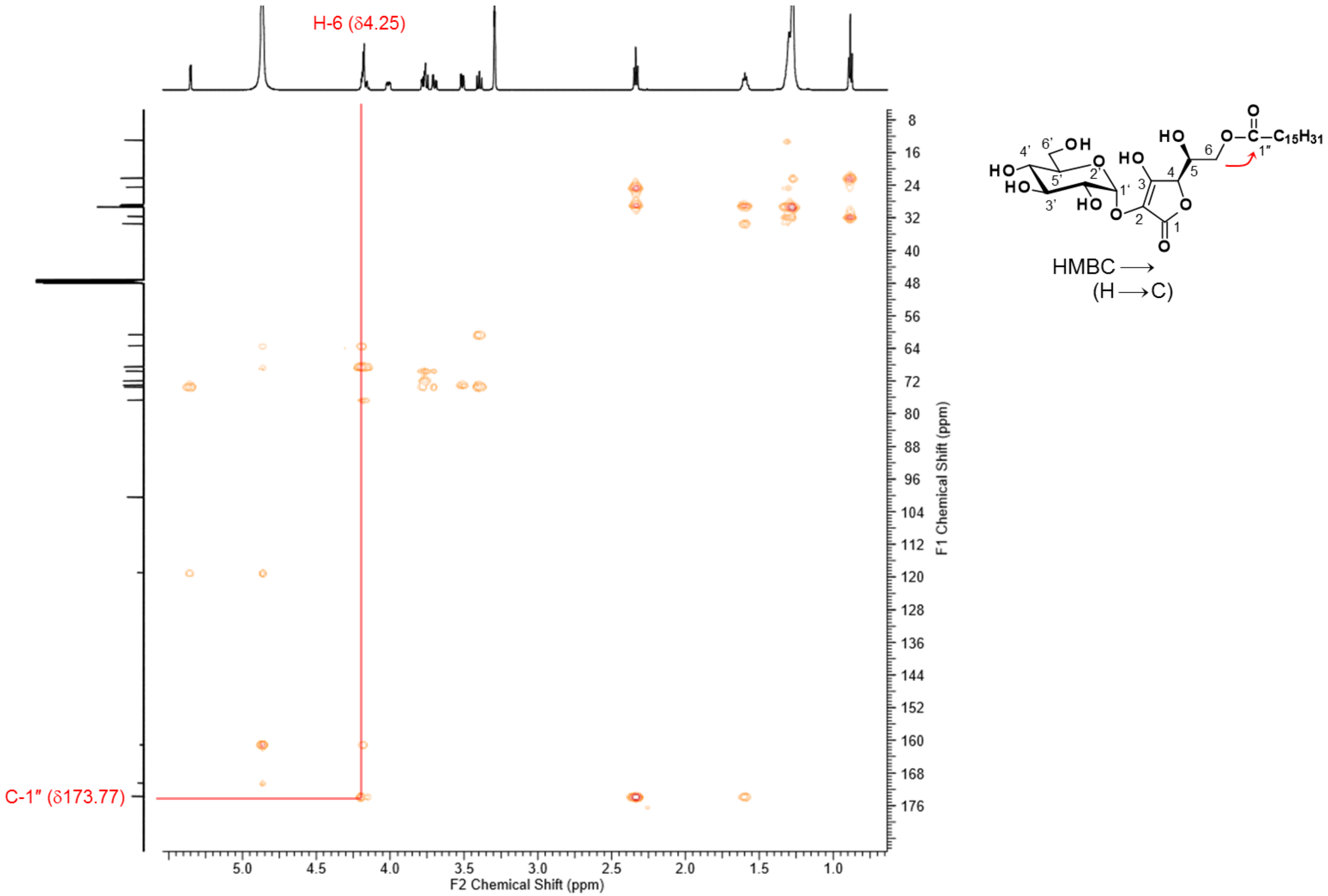

Figure S6. HMBC spectrum of 6-sPalm-EA-2G

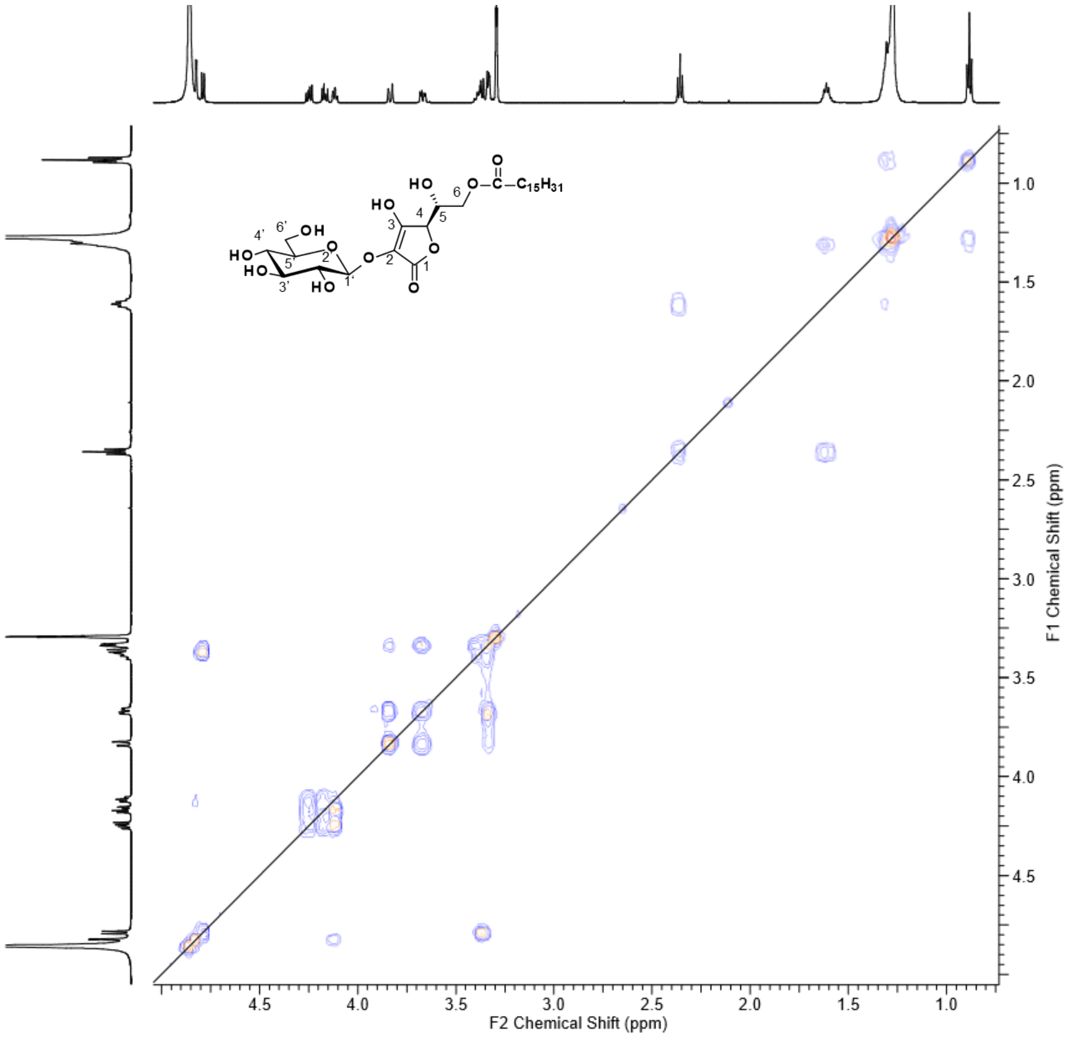

Figure S7. ^{1}H-^{1}H COSY spectrum of 6-sPalm-AA-2βG

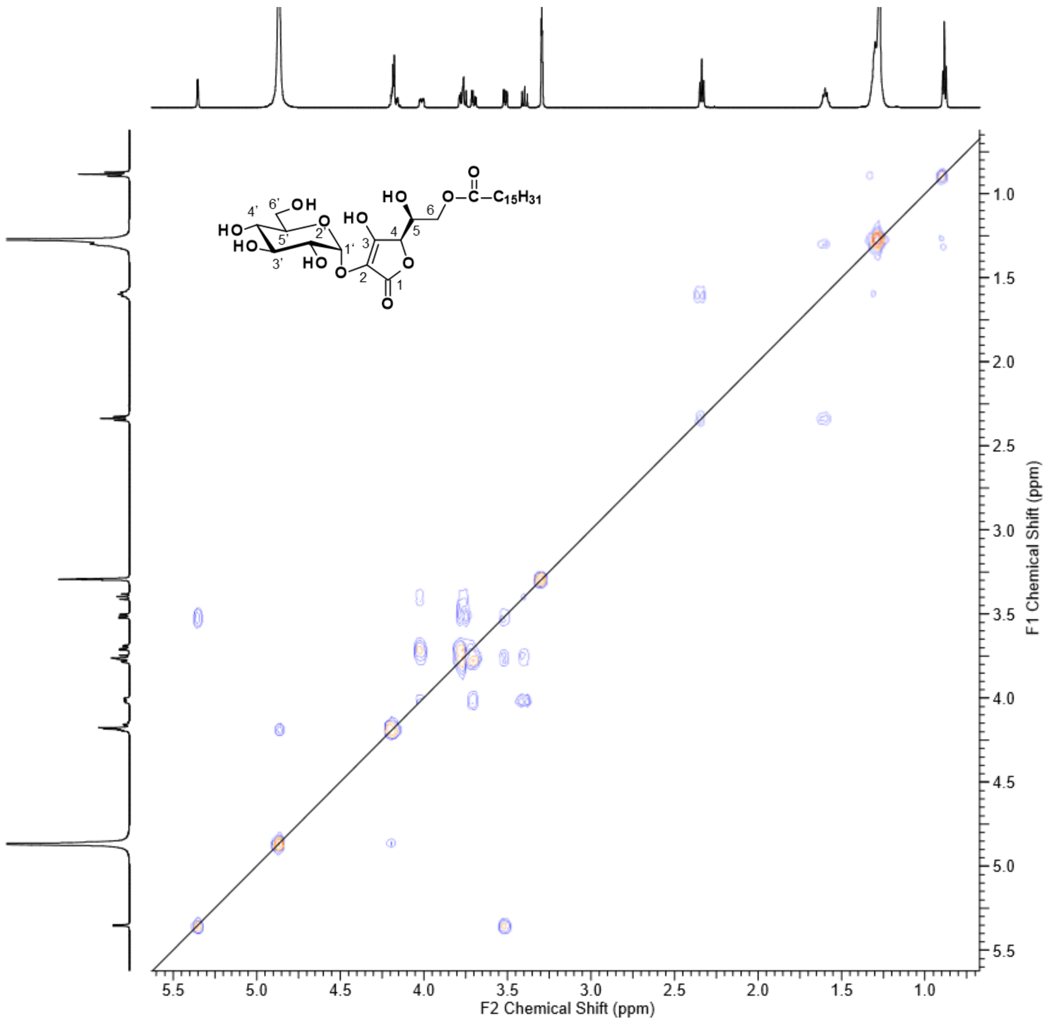

Figure S8. ^{1}H-^{1}H COSY spectrum of 6-sPalm-EA-2G
